# Identification and characterization of microRNAs in tree peony during chilling induced dormancy release by high-throughput sequencing

**DOI:** 10.1038/s41598-018-22415-5

**Published:** 2018-03-14

**Authors:** Yuxi Zhang, Yanyan Wang, Xuekai Gao, Chunying Liu, Shupeng Gai

**Affiliations:** College of Life Sciences, Qingdao Agricultural University, Key Lab of Plant Biotechnology in Universities of Shandong Province, Changcheng Road 700, Qingdao, China

## Abstract

Tree peony, one of the most valuable horticultural and medicinal plants in the world, has to go through winter to break dormancy. Growing studies from molecular aspects on dormancy release process have been reported, but inadequate study has been done on miRNA-guided regulation in tree peony. In this study, high-throughput sequencing was employed to identify and characterize miRNAs in three libraries (6 d, 18 d and 24 d chilling treatments). There were 7,122, 10,076 and 9,097 unique miRNA sequences belonging to 52, 87 and 68 miRNA families, respectively. A total of 32 conserved miRNAs and 17 putative novel miRNAs were identified during dormancy release. There were 771 unigenes as potential targets of 62 miRNA families. Total 112 known miRNAs were differentially expressed, of which 55 miRNAs were shared among three libraries and 28 miRNAs were only found in 18 d chilling duration library. The expression patterns of 15 conserved miRNAs were validated and classified into four types by RT-qPCR. Combining with our microarray data under same treatments, five miRNAs (*miR156k*, *miR159a*, *miR167a*, *miR169a* and *miR172a*) were inversely correlated to those of their target genes. Our results would provide new molecular basis about dormancy release in tree peony.

## Introduction

Tree peony (*Paeonia suffruticosa* Andrews) is one of the earliest and most well-known horticultural and medicinal plants in the world. Since flower buds of tree peony must go through a period of bud dormancy before bud sprouting in spring, the common adopted measure in agriculture is breaking dormancy by artificial chilling treatment for forcing culture. According to Lang and Martin^1^, the dormancy in tree peony belongs to endo-dormancy similar to some temperate fruit plants like grape, apple, peach, kiwifruit and so on. A sufficient chilling duration during winter is the main mean to break dormancy and induce growth in the following spring by appropriate warmer temperature^2^.

Release of dormancy physiological status was controlled through cooperation of large number of genes in woody plants^3^. Liu *et al*. obtained 190 significantly differentially expressed genes associated with bud dormancy in pear^4^. In Chinese cherry, totally 1,644 significantly differentially expressed genes were identified based on RNA-seq transcriptome^5^. To discover transcriptional pathways associated with dormancy release in *Prunus persica*, Fu *et al*. explored the chilling-dependent expressions of 11 genes involved in endoplasmic reticulum stress and the unfolded protein response signal pathways^6^. Yordanov *et al*. suggested that EARLY BUD-BREAK 1 (EBB1) have a major and integrative role in reactivation of meristem activity after winter dormancy in poplar trees^7^. In tea, sequence and gene ontology analysis of 3,500 clones associated with dormancy were analyzed^8^. In recent years, growing studies from molecular aspects on tree peony endo-dormancy release process have been reported, such as Huang *et al*. screened 31 unigenes associated with dormancy release in tree peony by SSH (suppression subtractive hybridization)^9^. Gai *et al*. obtained over 15,000 high quality unigenes by RNA sequencing during chilling requirement fulfillment through Roche 454 GS FLX platform^10^, of which 3,174 genes were significantly differentially expressed during endo-dormancy release in tree peony^11^. More recently, Zhang *et al*. obtained 20 differentially expressed protein spots (*P* < 0.05) during dormancy release by combination of two-dimensional gel electrophoresis (2-DE) and matrix-assisted laser desorption/ionisation time of flight/time of flight mass spectrometry (MALDI-TOF/TOF MS)^12^. In addition, Zhang *et al*. found a MADS-box member (SUPPRESSOR OF OVEREXPRESSION OF CONSTANS, *PsSOC1*) that not only accelerates flowering, but also promotes dormancy release in tree peony^13^. However, endo-dormancy mechanisms in tree peony are still unclear despite many efforts.

Eukaryotic gene expression is mainly regulated at the transcriptional and post-transcriptional levels. MicroRNAs (miRNAs) are a specific class of small non-coding RNA of commonly 19–24 nucleotides involved in post-transcriptional gene expression regulation^14^. The mature miRNAs negatively regulate gene expression through complementary binding to the opening reading frame (ORF) or UTR regions of specific target genes. In plants, miRNAs generally interact with their targets through near-perfect complementarily, which leads to gene silencing by endonucleolytic cleavage or translational inhibition^15–17^. Recent studies indicate that miRNAs play important roles in plant developments including organ separation, leaf development and polarity, lateral root formation, floral organ identity and reproduction, etc.^18–21^. Zhang *et al*. identified differentially expressed miRNAs responding to cold stress in tea^22^. Jeyaraj *et al*. analyzed the expression pattern of tea miRNAs in active and dormant bud using stem-loop pulse RT-qPCR method^23^. In poplar, ptr-miR169 was found to repress *ptrHAP2* at the level of post-transcription during vegetative bud dormancy period^24^. In tree peony, some of conserved and novel miRNAs were identified under copper stress^25^. However, no miRNAs have been reported especially during chilling endo-dormancy release in tree peony.

In this study, we aimed to identify and characterize miRNAs by high-throughput sequencing technology in tree peony during the period of bud dormancy release after 6 d, 18 d and 24 d chilling requirement fulfilling, which included three physiological status, endo-dormancy, endo-dormancy release and eco-dormancy^10^. Our results increase the available information on miRNA-guided regulation mechanism and physiological changes during chilling induced dormancy release in tree peony.

## Results

### Deep sequencing of Paoenia ostii sRNAs

**Figure 1:**
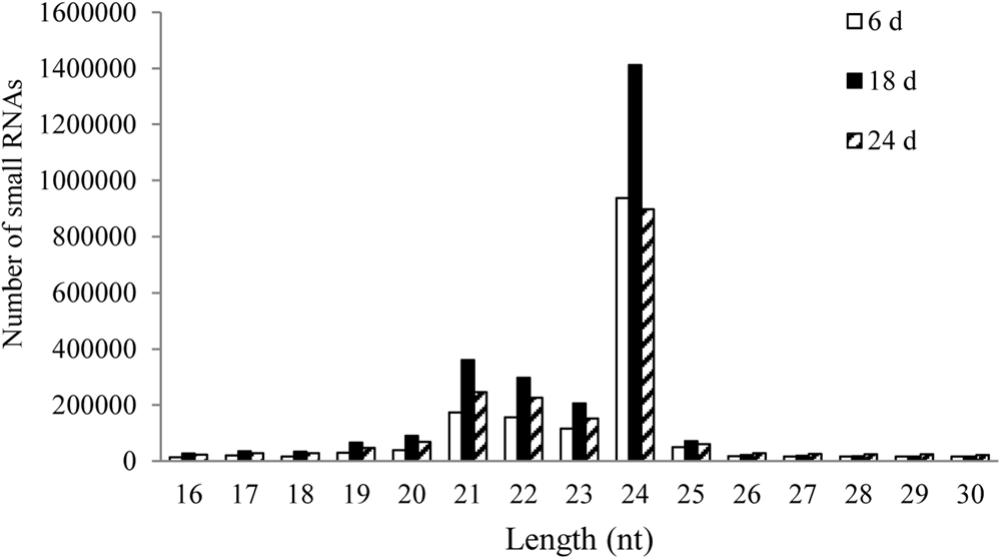
Length distribution of small RNAs in three libraries from tree peony buds after 6 d, 18 d and 24 d chilling treatments.

To investigate small RNA expression profiles in *Paoenia ostii* during physiological dormancy stages based on the results of morphologic observations^11^, three small RNA libraries of flower buds were constructed after 6 d, 18 d and 24 d chilling enduring. For each library, small RNAs were collected, pooled together and sequenced. A total of 19,762,599 reads with lengths of 16 bp to 30 bp were obtained from the three libraries, and average 3.8 million (range: 2.69–5.05 million) clean small RNA reads were acquired from each library after removing adapters and low-quality reads. The average number of unique reads per library was 2.06 million ranging from 1.63 to 2.68 million (Table 1). Most obtained sRNA sequences were 21–24 nt in all of the three libraries, of which 24 nt long sRNAs were the most abundant, accounting for approximately 52.3% on average (Fig. 1).

**Table 1:** Statistics of small RNA sequence reads.

Different treatments Title	6 d		18 d		24 d	
	Number	percent	Number	percent	Number	percent
Total Tags number	5,338,004	100%	8,092,580	100%	6,332,015	100%
Average quality < 13 Tags	1,469,084	27.52%	1,577,909	19.50%	1,224,509	19.34%
Length < 16	74,930	1.4%	204,694	2.53%	212,955	3.36%
Length > 30	615,543	11.53%	427,666	5.28%	507,468	8.01%
Clean number	2,686,857	50.33%	5,047,088	62.37%	3,770,180	59.54%
Unique number	1,629,348	30.52%	2,683,551	33.16%	1,895,066	29.93%

Clean data were searched against Rfam databases to annotate sRNAs, and known miRNAs were identified by alignment to sequences in miRBase 20.0 with no mismatch. siRNAs, ribosomal RNAs (rRNAs), tRNAs, snRNAs and snoRNAs were annotated by BLASTn to NCBI Genbank database and Rfam database. In order to eliminate the possibility of degraded mRNAs in three libraries, we aligned them through intron/exon alignment with unigenes in tree peony cDNA libraries^11^. The remaining unannotated sRNAs were used to predict novel miRNAs and potential miRNA seeds (Table 2). It is noticeable that the miRNAs represented 19.62% of the total sRNA in 6 d chilling treatments, but only 14.01% and 14.78% in 18 d and 24 d chilling treatments, which may as a result of many genes associated with endo- and eco-dormancy release are activated during dormancy release. There are about more 9,000 unique miRNAs at the physiological stage of dormancy release and eco-dormancy than at the status of dormancy, which indicates that the miRNA populations in flower buds after dormancy release are more diversified, as well as biological processes are more complex.

**Table 2:** Annotations of sRNAs against Rfam database and Unigenes.

Type	6 d				18 d				24 d			
	Unique	Percent (%)	Total	Percent (%)	Unique	Percent (%)	Total	Percent (%)	Unique	Percent (%)	Total	Percent (%)
Exon_antisense	94,368	32.25	260,542	26.46	137,004	26.46	493,402	29.67	118,471	29.84	419,569	26.77
Exon_sense	84,011	28.71	173,996	17.67	124,042	17.67	371,326	22.33	102,616	25.84	244,283	15.58
miRNA	7,122	2.434	193,210	19.62	10,076	19.63	232,971	14.01	9,097	2.29	231,665	14.78
rRNA	59,554	20.35	171,166	17.38	80,480	17.39	280,605	16.87	98,087	24.70	390,132	24.89
tRNA	11,327	3.87	47,986	4.87	15,948	4.87	44,297	2.66	15,367	3.87	51,773	3.3
snoRNA	8,995	3.07	26,961	2.73	16,174	2.74	55,757	3.35	12,911	3.25	57,613	3.68
snRNA	4,485	1.53	7,182	0.72	9,327	0.73	16,811	1.01	6,373	1.61	11,609	0.74
unannotated	22,737	7.77	103,457	10.5	45,640	10.51	167,553	10.01	34,122	8.59	160,894	10.26
Total	285,997	100	984,500	100	429,269	100	1,662,722	100	388,585	100.00	1,567,538	100

### Nucleotide Preference of 19–24 nucleotide small RNAs

**Figure 2:**
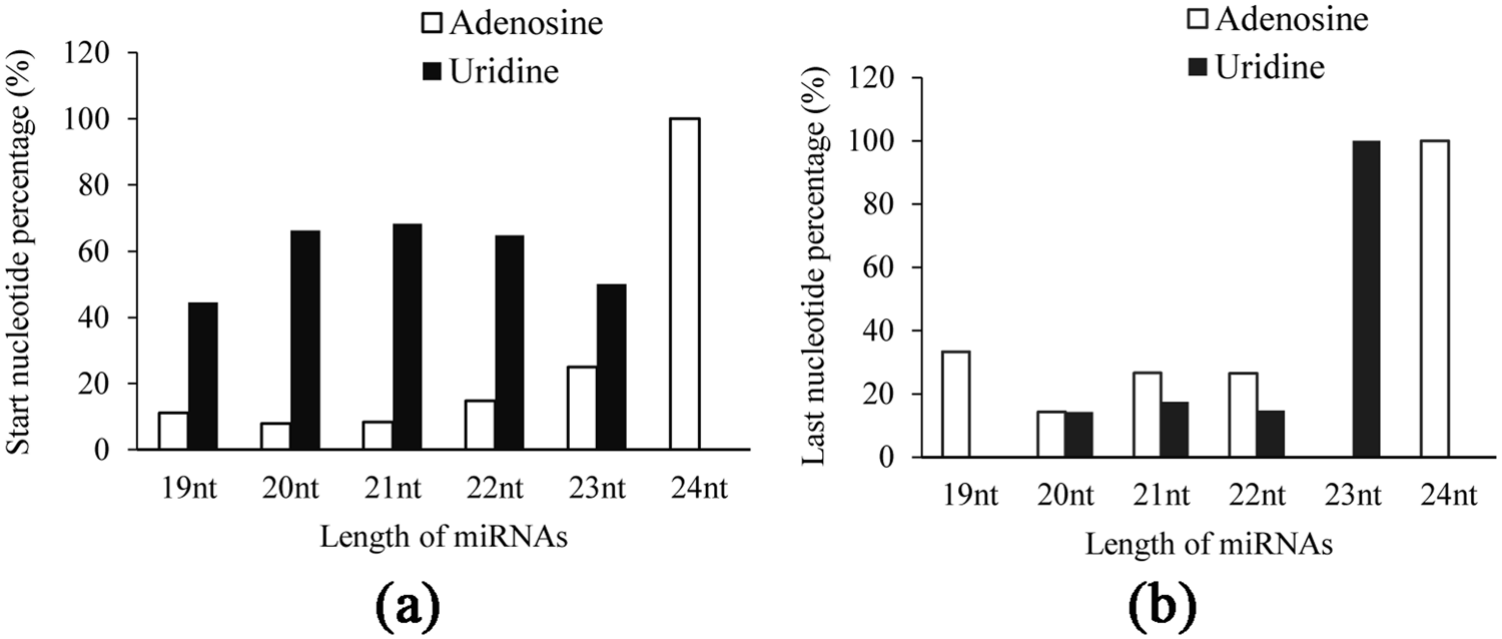
Nucleotide preference of small RNAs. (**a**) Percentage of adenosine or uridine at the start position of 19 to 24-nucleotide (nt) small RNAs. (**b**) Percentage of adenosine or uridine at the last position of 19 to 24-nucleotide (nt) small RNAs.

Previous studies have shown that most miRNA sequences start with uridine (U), whereas the majority of 24-nucleotide siRNAs have adenosine (A) as their 5′ first nucleotide in plants^26–29^. In our result, the same trends were observed among cloned tree peony small RNAs, about 67.1% of small RNAs sequences started with uridine, and all 24-nt siRNAs had start-nucleotide preference with adenosine (Fig. 2b). Besides, we found that about 45.5% of total small RNAs also had a clear preference for adenosine as the last nucleotide, of which all 23-nt had a clear preference for uridine and all 24-nt for adenosine as the last nucleotide (Fig. 2b). In order to investigate whether the last nucleotide preference from 19 nt to 24 nt small RNAs also exist in other model plants like Arabidopsis, we downloaded 427 Arabidopsis small RNA deep sequencing datasets from miRBase database (http://www.mirbase.org/ftp.shtml) and analyzed their nucleotide compositions (Supplementary Figure S1). In all 427 Arabidopsis datasets, strong last nucleotide preference for uridine was observed in 23 and 24 nt small RNAs, indicating that difference of nucleotide preference might exist among species.

### Identification of conserved and novel miRNAs in tree peony

**Figure 3:**
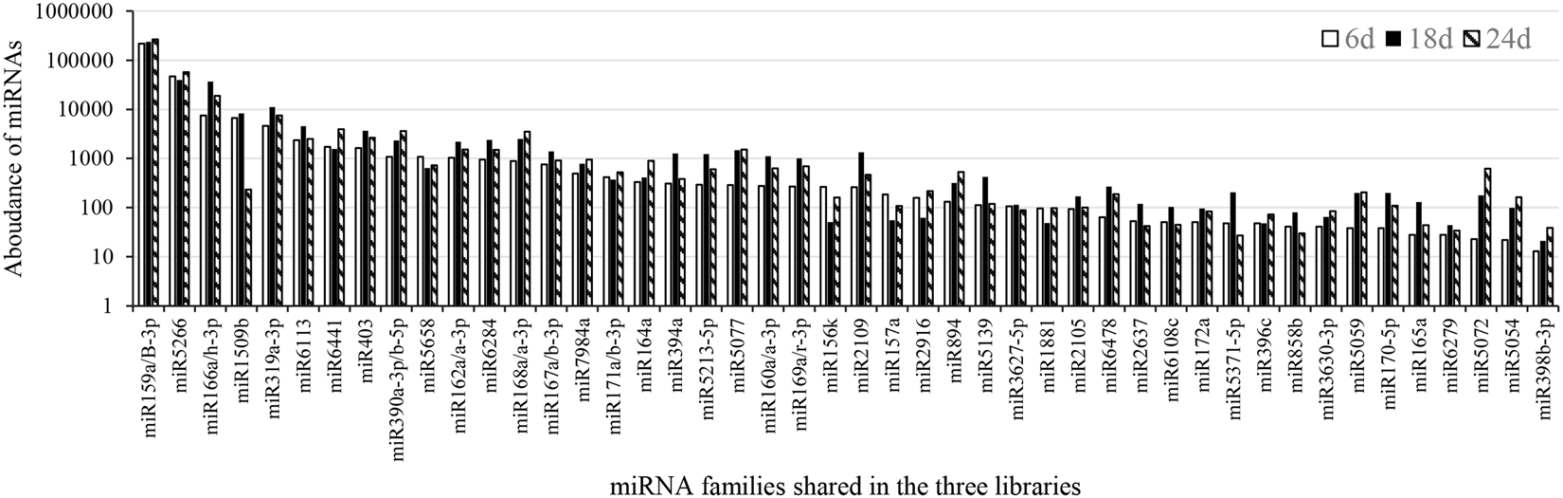
Abundance of most conserved miRNA families in three libraries from tree peony buds after 6 d, 18 d and 24 d chilling treatments.

To identify conserved miRNAs in tree peony, but its genome is not available, known plant miRNAs (miRNA precursors and mature miRNAs) registered in miRBase 21.0 were used as reference for miRNA mapping. Clean data that aligned to known miRNAs allowing two mismatches and had no less than 5 reads per million (RPM) in at least one library were regarded as conserved miRNAs. In three libraries, total 112 known miRNAs belonging to 99 miRNA families were identified in the three libraries, of which there were 7,122, 10,076 and 9,097 unique miRNA sequences belonging to 52, 87 and 68 miRNA families, respectively (Additional file 1, Table 3). Of which, there was 32 conserved miRNAs (Table 4). In our data, 15 miRNAs sequences were found anchored in the 5p-arm and 17 miRNAs anchored in the 3p-arm (Addition file 1, Table 4). Unexpectedly, one less-conserved miRNA (*miR5072*) was obtained, which was previously found only in monocots^30^. Furthermore, there were 55 miRNAs belonging to 46 miRNA families shared in the three libraries (Fig. 3), and the most abundant miRNA identified by sequence homology was *PsmiR159* with more than 500,000 actual sequencing reads, accounting for approximately 69% of the total conserved miRNA reads, following by *PsmiR5266* with more than 100,000 actual sequencing reads, *PsmiR166*, *PsmiR319*, *PsmiR1509* with more than 10,000 actual sequencing reads, and *miR398* showed the minimum amount (Table 4). At the same time, the frequencies of miRNAs read varied from 8 (*PsmiR2111a-5p*) to 709,087 (*PsmiR159a*), which indicate that miRNAs displayed drastically different expression level in tree peony during dormancy release (Fig. 3). After normalization, more than half of the conserved miRNAs were less than 100 times. In addition, the relative abundance of certain members within same miRNA family varied widely (Table 4). For instance, the normalized number of *PsmiR167a* was 255, but that for *PsmiR167b* was only 12. Furthermore, the normalized reads of different members in three treatments were significant different, for example, the abundances of members in *miR159* (*miR159a* and *miR159b-3p*) ranged from 18,716, 180 (6 d), 20,170, 496 (18 d) to 22,752 and 218 (24 d) reads in three libraries, respectively (Table 4). These results suggest that members showed different expression trends within same miRNA family, probably because their expressions are development-stage specific or either induced or suppressed during dormancy release in tree peony.

**Table 3:** Summary of small RNA deep sequencing data.

Libraries	No. of sequences generated^a^	No. of non-redundant sequences^a^	No. of sequences with perfect matches to the miRBase	Unique miRNA number	Family number
6 d	2,686,857	1,629,348	193,210	7,122	52
18 d	5,047,088	2,683,551	232,971	10,076	87
24 d	3,770,180	1,895,066	231,665	9,097	68

^a^Lengths between 16–30 nt.

**Table 4:** Known miRNAs identified from tree peony flower bud after different chilling treatments.

Family	miRNA IDs	sequences	Actual sequencing reads /Normalized sequencing reads			zma	ath	osa	vvi	ptc
			6d	18d	24d	zma	ath	osa	vvi	ptc
**well-conserved**										
miR156	*PsmiR156k*	UGACGGAGAGAGAGAGCACAC	263/23	51/4	161/14	0	0	0	0	0
	*PsmiR156f-3p*	GCUCACUCUCUAUCUGUCACC	0/0	23/2	0/0	0	0	0	0	0
miR159	*PsmiR159a*	UUUGGAUUGAAGGGAGCUCUA	215,316/18,716	232,033/20,170	261,738/22,752	+	0	+	0	0
	*PsmiR159b-3p*	UAUUGGAGUGAAGGGAGCUCC	2,067/180	5,704/496	2,505/218	+	+	0	+	+
miR160	*PsmiR*16*0a*	UGCCUGGCUCCCUGUAUGCCA	216/19	1,071/93	577/50	+	+	0	0	0
	*PsmiR160a-3p*	GCGUAUGAGGAGCCAAGCAUA	60/5	45/4	49/4	0	0	0	0	0
miR162	*PsmiR162-3p*	UCGAUAAACCUCUGCAUCCA	551/48	1,236/107	913/79	NA	0	0	0	0
	*PsmiR162a*	UCGAUAAACCUCUGCAUCCAG	489/43	954/83	602/52	0	0	0	0	0
	*PsmiR162b*	UGCCUGGCUCCCUGUAUGCCA	112/10	4/0	740/64	0	0	0	0	0
miR164	*PsmiR164a*	UGGAGAAGGGGAGCACGUGCA	332/29	409/36	900/78	+	++	+	0	0
	*PsmiR164b*	UGGAGAAGCAGGGCACAUGCU	2/0	10/1	740/64	+	++	+	0	0
	*PsmiR164c*	UGGAGAAGCAGGGCACGUGCU	524/46	8/1	2/0	0	+	0	+	+
miR166	*PsmiR166a*	UCGGACCAGGCUUCAUUCCCC	3,665/319	19,051/1,656	7,933/690	NA	NA	NA	NA	NA
	*PsmiR166h-3p*	UCUCGGACCAGGCUUCAUUCC	3761/330	17,903/1,556	11,027/959	0	0	0	0	0
miR167	*PsmiR167a*	UGAAGCUGCCAGCAUGAUCUGA	714/63	1,339/116	884/77	0	0	0	0	0
	*PsmiR167b-3p*	GGUCAUGCUCUGACAGCCUCACU	41/4	56/5	33/3	0	0	0	0	0
miR168	*PsmiR168a*	UCGCUUGGUGCAGGUCGGGAA	738/64	2,375/206	3,210/279	++	0	++	0	0
	*PsmiR168a-3p*	CCCGCCUUGCAUCAACUGAAU	151/13	140/12	295/26	++	0	++	0	0
miR169	*PsmiR169a*	CAGCCAAGGAUGACUUGCCGA	50/4	170/15	77/7	0	0	0	0	0
	*PsmiR169r-3p*	GCAAGUUGUCUUUGGCUACA	218/19	837/73	615/53	0	0	0	0	0
miR171	*PsmiR*17*1a*	UGAUUGAGCCGCGCCAGUAUC	277/24	234/20	346/30	0	0	0	0	+
	*PsmiR171b-3p*	UUGAGCCGCGUCAAUAUCUCU	141/12	136/12	179/16	+	+	+	0	++
miR172	*PsmiR172a*	AGAAUCUUGAUGAUGCUGCAU	51/4	96/8	84/7	0	0	0	+	0
	*PsmiR172a-3p*	GCAGCGUCCUCAAGAUUCACA	2/0	6/1	10/1	NA	NA	NA	NA	NA
miR319	*PsmiR319a*	UUGGACUGAAGGGAGCUCCCU	0/0	4,519/393	10,486/911	+	0	+	0	0
	*PsmiR319a-3p*	UUGGACUGAAGGGAGCUCCC	4,587/403	11,200/974	7,432/646	NA	0	NA	0	NA
	*PsmiR319i*	UUGGACUGAAGGGGGCUCCC	1,408/122	0/0	0/0	0	0	NA	0	NA
miR390	*PsmiR390a-3p*	CGCUAUCCAUCCUGAGUCUCA	26/2	139/12	136/12	++	+	++	+	0
	*PsmiR390b-5p*	AAGCUCAGGAGGGAUAGCACC	1,062/92	2,193/191	3,466/301	0	0	0	0	0
miR394	*PsmiR394a*	UUGGCAUUCUGUCCACCUCC	307/27	1,266/110	385/33	0	0	0	0	0
miR396	*PsmiR396b-3p*	GCUCAAGAAAGCUGUGGGAAA	0/0	17/1	18/2	++	NA	NA	NA	NA
	*PsmiR396c*	UUCAAGAAAGUCGUGGGAGA	48/4	48/4	73/6	++	++	++	++	+
**Less-conserved**										
miR5266	*PsmiR5266*	CGGGGGACUGCUCGGGCC	46,659/4,056	39,563/3,439	57,262/4,978	NA	NA	NA	NA	NA
*miR4414*	*PsmiR4414a-5p*	AGCUGCUGACUCGUUCAUUCA	0/0	23/2	0/0	NA	NA	NA	NA	NA
	*PsmiR4414b*	UGUGAAUGAUGCGGGAGACAA	70/6	0/0	102/9	NA	NA	NA	NA	NA
miR403	*PsmiR403*	UUAGAUUCACGCACAAACCCA	1,629/142	3,659/318	2,662/231	NA	++	NA	++	++
miR5054	*PsmiR5054*	GCCCCACGGUGGGCGCCA	22/2	99/9	162/14	NA	NA	NA	NA	NA
miR5059	*PsmiR5059*	UCCUGGGCAGCAACACCA	38/3	200/17	206/18	NA	NA	NA	NA	NA
miR5077	*PsmiR5077*	UUCACGUCGGGUUCACCA	288/25	1,483/129	1,529/133	NA	NA	NA	NA	NA
miR5139	*PsmiR5*13*9*	AACCUGGCUCUGAUACCA	113/10	425/37	119/10	NA	NA	NA	NA	NA
miR5213	*PsmiR5213-5p*	UGCGUGUGUCUUCACCUCUGA	293/25	1,228/107	606/53	NA	NA	NA	NA	NA
miR5371	*PsmiR5371-5p*	UUGGAAUCUAGUCGACUCAGAC	48/4	205/18	27/2	NA	NA	NA	NA	NA
miR5658	*PsmiR5658*	AUGAUGAUGAUGCUGAGAC	1,085/94	640/56	723/63	NA	++++	NA	NA	NA
miR6108c	*PsmiR6108c*	AAUCGUAAGAAGAAUGCUGAAGCC	51/4	103/9	45/4	NA	NA	NA	NA	NA
miR6113	*PsmiR6113*	UGAAACUCAAGAAAACGUCG	2,367/206	4,552/396	2,504/218	NA	NA	NA	NA	NA
miR6279	*PsmiR6279*	UAGAAAGUAAUUCCAUGACACC	28/2	44/4	34/3	NA	NA	NA	NA	NA
miR6284	*PsmiR6284*	UACUUGGACCCUGAAUGAAGAUU	954//83	2,398/208	1,505/131	NA	NA	NA	NA	NA
miR6441	*PsmiR6441*	AAUUGACGGAAGGGCACA	1,738/151	1,553/135	3,920/341	NA	NA	NA	NA	++++
miR6478	*PsmiR6478*	CCGACCUUAGCUCAGUUGGUAGA	64/6	270/23	187/16	NA	NA	NA	NA	+
miR7984a	*PsmiR7984a*	UCCGACUUUGUGAAAUGACUU	492/43	778//68	953/83	NA	NA	NA	NA	NA
miR858b	*PsmiR858b*	UUCGUUGUCUGUUCGACCUUG	41/4	80/7	30/3	NA	0	NA	NA	NA
miR894	*PsmiR894*	UGUUCGUUUCACGUCGGGUUCACCA	131/11	320/28	530/46	NA	NA	NA	NA	NA
miR6300	*PsmiR6300*	GUCGUUGUAGUAUAGUGG	10,410/905	0/0	0/0	NA	NA	NA	NA	NA
miR398	*PsmiR398b-3p*	UUGUGUUCUCAGGUCACCCCU	13/1	21/2	39/3	NA	NA	0	NA	0
miR5072	*PsmiR*5072	AACGACUCCCCAGCAGAGUCGCC	23/2	178/15	622/54	0	NA	0	NA	+

0 represents no mismatch, + represents one mismatch, ++ represents tow mismatches, and so on. zma, *Zea mays*; ath, *Arabidopsis thaliana*; osa, *Oryza sativa*; vvi, *Vitis vinifera*; ptc, *Populus trichocarpa*.

**Figure 4:**
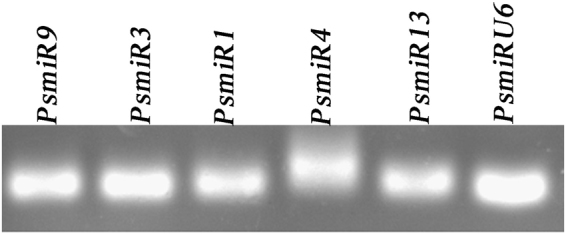
Reverse transcript PCR (RT-PCR) electrophoresis results for expression identification of novel miRNAs in flower buds after 18 d chilling treatments. In total, 5 of 17 novel miRNAs were confirmed by Reverse transcript PCR (RT-PCR) with 40 cycle-amplification. The sizes of PCR products were about 100 bp. *PsU6* was used as positive control.

Based on the miRNA annotation criteria^31^, novel miRNAs could be predicted by mapping the remaining non-annotated sRNAs to *Populus* genome. In our data, seventeen novel miRNAs were obtained and named as *PsmiR1* to *PsmiR17* (Table 5). All precursors of novel miRNAs had regular stem-loop structures and the predicted hairpin structures. To investigate the conservation of these 17 novel miRNAs in other plant species including *Malus domestica*, *Physcomitrella patens* and *Populus trichocarpa*, they were used to perform BLAST searches against miRBase databases. *PsmiR5, PsmiR7* and *PsmiR16* matched genomes of other plant species (Table 5). Reverse transcript PCR (RT-PCR) was performed to validate the expression of some new predicted miRNAs in flower buds after chilling treatments. The primer sequences were listed in Supplementary Table S1. We found five of the 17 predicted miRNAs including *PsmiR9, PsmiR3, PsmiR1, PsmiR*4 and *PsmiR13* expressed in tree peony flower buds (Fig. 4). *PsU6* was amplified as a positive control. We found that these novel miRNAs could be detected in flower buds after 18 d chilling treatments.

**Table 5:** Candidate novel miRNAs in tree peony.

novel miRNA	mature sequence (5′-3′)	other species’ ID in miRbase	MFE (kcAl/mol)	MFEI	Predicted precursors	p-value	Predicted target ID	Normalized miRNA abundance		
								6 d	18 d	24 d
*PsmiR1*-5p	AGGGACTCCTTTCACTCCACT	—	−81.9	1	JI448260:47.0.214:+	0.99	—	7,624.35	9,555.42	23,848.02
*PsmiR2*-5p	CATACTTCTGGATAACG	—	−11.2	0.4	JI455606:1198..1247:+	0	JI455773	0	4.78	2.65
*PsmiR3*-5p	GGTGGACTGCTCGAGCC	—	−27.8	0.9	JI443786:93..136:-	0.99	JI450527	23,0084.13	159,333.51	223,257.97
*PsmiR 4*-3p	TATGAGACTTGGACGAGGCAC	—	−37.9	1	JI451506:405..481:-	0.99	JI445930	3,384.18	2,348.2	2,987.29
*PsmiR 5*-5p	AGAGAATTGAAGATGAGCACCT	ppt-miR1023b-3p	−41.2	0.7	ContiG02457:19..227:-	0	JI446191	0	0	2.65
*PsmiR 6*-3p	ATCTCTTTGAGCTGCAAGAAGGCC	—	−66.4	0.5	JI458593:131..405:-	0	JI458593	0	0	2.65
*PsmiR 7*-3p	AAGCCATGGATGAAGCTAT	ptc-miR169y	−82	0.7	JI448255:1023..1305:-	0	JI450838	0	4.78	0
*PsmiR 8*-5p	TGTACTACAGGGTAGGAAAGA	—	−24.3	0.9	JI456175:234..311:+	0.99,	JI452932	79.63	45.43	108.48
*PsmiR 9*-5p	CGGTGGACTGCTCGAGCCG	—	−28.9	0.9	JI443786:93..137:-	0	JI443786	378,494.16	410,756.3	374,225.73
*PsmiR 10*-5p	AGCCTTCTTTGGGTTGCGACC	—	−73.2	1.3	JI448950:74..188:-	0	JI448950	23.89	253.47	58.21
*PsmiR 11*-5p	AGCTTTTGTATGTTCTCCGTTA	—	−57.7	0.6	JI452338:1477..1738:+	0	—	0	2.39	0
*PsmiR 12*-5p	GGTGGATGTATGAACCCAGCCT		−47.6	0.6	JI454613:413..564:+	0	JI453131	0	2.39	0
*PsmiR 13*-5p	TTGTTTGAATTCTTGCAACAGA	—	−63.9	1.5	JI444316:158..242:+	0	JI444316	1,282.01	4,421.41	828.18
*PsmiR 14*-3p	CGACTGGGAAGGATTGGGGA	—	−80	0.6	JI454686:1279..1546:-	0	JI454686	0	0	2.65
*PsmiR 15*-5p	AGGGCATGTCCATGGGCTCT	—	−49.4	0.5	JI458732:712..921: -	0	JI458732	0	2.39	0
*PsmiR 16*-3p	AGAAGAGAAGAGAGAGGA	mdm-miR169e	−29.9	0.6	JI452660:25..140: -	0	JI457551	0	0	2.65
*PsmiR 17*-3p	CCAAGTTAAGCTCGGCGAG		−10	0.4	JI450973:261..299:+	0	JI453625	0	2.39	0
*PsmiR 13*-3p	TGTTGCAGAATTCAAACAAA	—	−63.9	1.5	JI444316:158..242:+	0	JI444316	557.39	2,529.94	494.79

ppt: *Physcomitrella patens*; ptc: *Populus trichocarpa;* mdm*: Malus domestica*.

We found 469 mature miRNAs could be aligned to other species’ precursors (precursors data from miRbase r21), mostly in *Glycine max* (36.7%), *Oryza sativa* (30.7%), and *Arabidopsis thaliana* (30.06%). The 17 precursors of novel miRNAs derived from tree peony transcriptome was listed in Table 5, only 1 precursors coded both 5p and 3p side mature miRNAs, others only possessed 1-side mature sequence.

### Prediction of miRNA targets in tree peony

**Figure 5:**
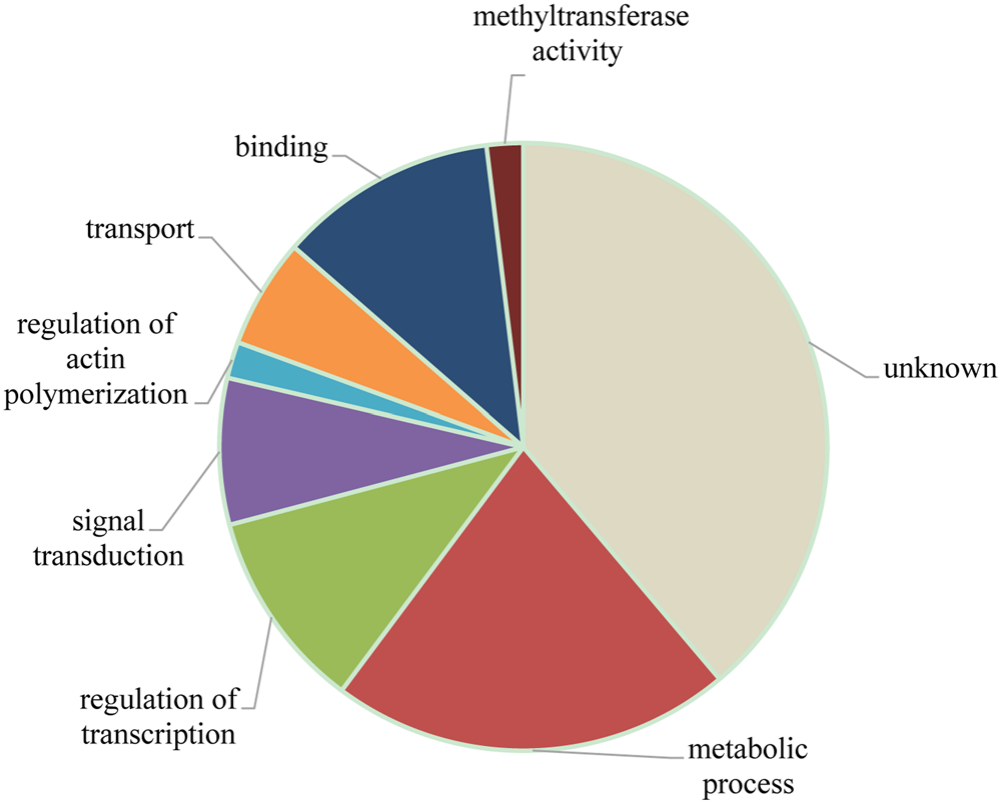
Go analysis of targets of known miRNAs in this study.

Previous study found that plant miRNA target sites mainly situate at opening reading frames (ORFs)^32^. To understand possible biological functions of the identified miRNAs in tree peony, we predicted the miRNA targets using the mRNA transcriptome of *Paoenia ostii* flower buds as a reference sequence since the genome of *Paoenia ostii* is not publicly available^10^. A total of 771 unigenes were predicted as potential targets of 62 known miRNA families (Additional file 2), and the majority of target proteins and corresponding annotations were shown in Table 6. Most miRNAs had more than one predicted target proteins, and some of the miRNAs have more than 10. Based on GO annotation, more than half of the predicted target genes were involved in biological process (metabolic process, regulation of transcription, signal transduction, transport and regulation of act polymerization) and molecular function (binding and methyltransferase activity) (Fig. 5). However, there were many conserved miRNAs target genes that had no functional annotation. Novel miRNAs targets were also predicted, but only two of them have been found target relationship with two unigenes (Table 5).

**Table 6:** Majority of the predicted target genes and corresponding annotation of known miRNAs in tree peony.

miRNA family	Target ID	Targets annotation	miRNA family	Target ID	Targets annotation
miR1509	JI451099	Peroxiredoxin	miR319	JI449827	AP2 domain-containing transcription factor
miR156	JI447102	DNMT2 (DNA METHYLTRANSFERASE-2)	miR319	JI447690	Polygalacturonase precursor
miR156	JI446831	SQUAMOSA promoter-binding protein-like	miR395	JI453154	beta galactosidase
miR159	JI446967	RAB6A; GTP binding/protein binding	miR396	JI445772	Chitin-inducible gibberellin-responsive protein
miR159	JI446401	asparagine synthetase	miR396	JI444318	glyceraldehyde 3-phosphate dehydrogenase
miR162	JI449996	ubiquitin	miR397	JI455403	lipoxygenase
miR164	JI458131	PID2 (PINOID2); ATP binding/protein kinase	miR414	JI453820	zinc finger protein
miR164	JI449546	dtdp-glucose 4-6-dehydratase	miR414	JI445308	phosphoesterase
miR166	JI446960	pentatricopeptide repeat-containing protein	miR414	JI444902	Phospho-2-dehydro-3-deoxyheptonate aldolase 1
miR167	JI458177	transmembrane protein	miR5059	JI445796	CAM7 (CALMODULIN 7); calcium ion binding
miR167	JI452318	trytophan synthase alpha subunit	miR5083	JI446932	COP1-interacting protein-related
miR167	JI451976	Serine/threonine-protein kinase PBS1	miR5658	JI455720	Serine/threonine-protein kinase SAPK10
miR168	JI451707	GRAS family transcription factor	miR5658	JI454156	dolichyl glycosyltransferase
miR169	JI454583	similar to Protein kinase	miR5658	JI451464	Stromal cell-derived factor 2 precursor
miR169	JI450321	Acetyl glucosaminyl transferase	miR6113	JI450180	Lactoyl glutathione lyase
miR169	JI447773	F-box family protein	miR6300	JI450877	3-dehydroquinate synthase
miR169	JI445119	calcium-dependent protein kinase	miR6300	JI450707	DNA damage checkpoint protein
miR171	JI449485	endoglucanase	miR6300	JI448956	ankyrin repeat domain protein
miR172	JI445772	Transcription factor GRAS	miR8175	JI444623	aminobutyrate aminotransferase
miR172	JI446524	AP2 domain-containing transcription factor	miR845	JI448900	cell division protein

**Figure 6:**
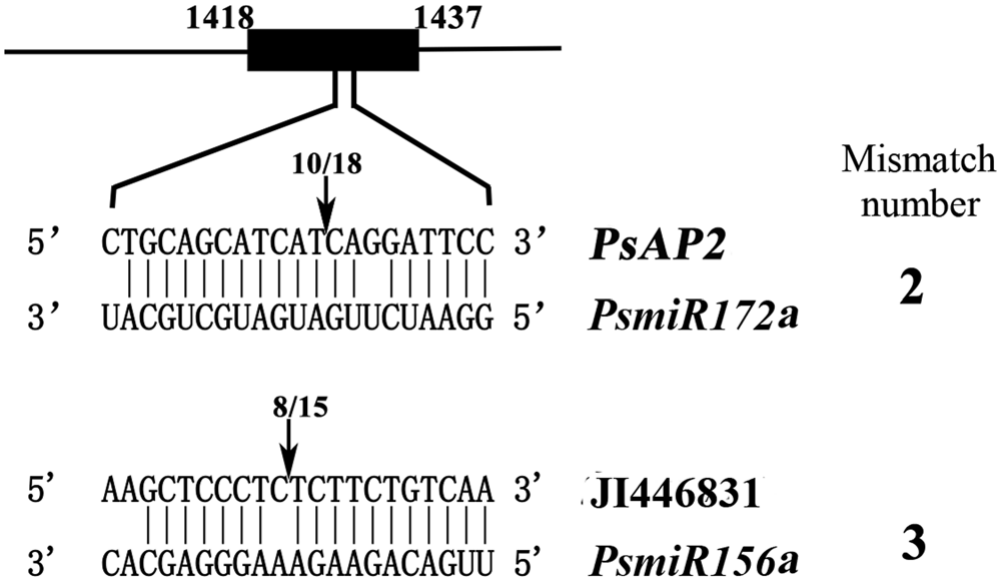
Validation of miRNA predicted targets by 5′ RLM-RACE in tree peony bud. Positions of the cleavage sites are indicated by arrows with the proportion of sequenced clones.

5′-RNA ligase-mediated (RLM)- RACEs were carried out to confirm the miRNA-guided cleavage and cleavage sites of predicted target transcripts. Squamosa-promoter-binding protein-like (SPL) family genes and APETALA2 (AP2) had been reported that they were the predicted targets of miR156 and miR172, respectively^15,33^. Our results showed that *PsAP2* (JI446524) could be cleaved at the site between bases 12 (T) and 13 (C) within the complementary region to *PsmiR172a* (Fig. 6). *PsSPL* (JI446831) could be cleaved by *PsmiR1*56 at the site between 10 (C) and 11 (T), which was also identified as a miRNA cleavage site in rose^33^.

### Expression profile of *Paoenia ostii* miRNAs during dormancy release

To identify miRNAs that were responsive during dormancy release, we compared miRNA expression level among three libraries. All the conserved candidate miRNAs with no less than 10 reads in each library were analyzed. Differentially expressed miRNAs that exhibited more than a 2-fold change were selected between each two treatments. There were 112 known miRNAs differentially expressed among three libraries (Additional file 3), and of which 55 miRNAs were shared among three libraries (See Additional file 3-shared miRNAs in three libraries). There existed 28 miRNAs (including *miR8126-3p*, *miR64*7*9*, *miR2949b*, *miR5057*, *miR6144*, *miR7743-3p*, *miR64*8*3, miR5083*, etc.) only in 18 d chilling duration library, which might play important roles in regulation dormancy release (Additional file 4). Based on the method of Audic and Man, the shared miRNAs among three libraries were normalized (Table 7). There were 11 down-regulated and 43 up-regulated miRNAs from 6 d to 18 d chilling treatment. Among them, *PsmiR5072* showed the highest degree of induction (7.7-fold), *PsmiR3*9*0a-3p*, *PsmiR5059*, *PsmiR170-5p*, *PsmiR166a*, *PsmiR2*10*9* and *PsmiR5077* were also clearly up-regulated (>5-fold). In addition, *PsmiR5519* was specifically and significantly induced during dormancy release. To further elucidate the potential regulatory roles of miRNAs in the transition from dormancy to eco-dormancy, we made a comparative analysis of miRNA expression between 18 d chilling and 24 d chilling. There were 31 down-regulated and 24 up-regulated miRNAs, among them *PsmiR1509b* was the most significantly down-regulated (>35-fold). Similar to the report in poplar^34^, eight known development-related miRNA families were also detected differentially expressed during dormancy release in our data, including *PsmiR164*, *PsmiR396*, *PsmiR168*, *PsmiR319*, *PsmiR171*, *PsmiR166*, *PsmiR156* and *PsmiR172*. These miRNAs mainly acted on cell proliferation (*miR164*, *miR396* and *miR319*)^28,35^, vascular development (*miR166*)^36^ and miRNA biogenesis (*miR168*)^37^. *PsmiR164* and *PsmiR168a* were continuously induced from dormancy to eco-dormancy stage, which were different from that in poplar^34^. *PsmiR166* was up-regulated during dormancy release and repressed in eco-dormancy stage, similar results were detected in poplar during chilling induced dormancy-release^34^. Members of the *PsmiR171* and *PsmiR166* families showed the same expression patterns, but distinct differences of expression levels were also observed within other families during the same process. For example, *PsmiR159a* was up-regulated from 18 d to 24 d chilling treatments, while *PsmiR159b-3p* was down-regulated (>2-fold). *PsmiR168a-3p* was repressed during dormancy release (from 6 d chilling to 18 d chilling treatments), but *PsmiR168a* was continuously induced, indicating that members from same miRNA family might play different roles during this process. In addition, there were 28 miRNAs detected only in 18 d chilling treatment library and 7 miRNAs (*PsmiR5227*, *PsmiR5665*, *PsmiR1886.1*, *PsmiR774b-5p*, *PsmiR4357*, *PsmiR1217-5p* and *PsmiR5224b*) only in 24 d chilling treatment library (Additional file 4), which might mainly function in the transition from dormancy to dormancy release stage and eco-dormancy stage, respectively.

**Table 7:** Differentiated expressions of shared miRNAs from tree peony flower bud after different chilling treatments.

miRNA	Dormancy release	Endo-dormancy	miRNA	Dormancy release	Endo-dormancy	miRNA	Dormancy release	Endo-dormancy
	18 d vs 6 d	24 d vs 18 d		18 d vs 6 d	24 d vs 18 d		18 d vs 6 d	24 d vs 18 d
*PsmiR* *5072*	7.73913	3.494382	*PsmiR* *159b* *-3p*	2.759555	−2.27705	*PsmiR* *167b* *-3p*	1.365854	−1.69697
*PsmiR* *390a* *-3p*	5.346154	−1.02206	*PsmiR* *6284*	2.513627	−1.59336	*PsmiR* *1509b*	1.239167	−35.2232
*PsmiR* *5059*	5.263158	1.03	*PsmiR* *894*	2.442748	1.65625	*PsmiR* *164a*	1.231928	2.200489
*PsmiR* *170* *-5p*	5.263158	−1.83486	*PsmiR* *319a* *-3p*	2.441683	−1.507	*PsmiR* *3627* *-5p*	1.084906	−1.29213
*PsmiR* *166a*	5.19809	−2.40149	*PsmiR* *403*	2.246163	−1.37453	*PsmiR* *159a*	1.077639	1.128021
*PsmiR* *2109*	5.153846	−2.88793	*PsmiR* *2637*	2.245283	−2.83333	*PsmiR* *396c*	1	1.520833
*PsmiR* *5077*	5.149306	1.031018	*PsmiR* *162* *-3p*	2.243194	−1.35378	*PsmiR* *171b* *-3p*	−1.03676	1.316176
*PsmiR* *160a*	4.958333	−1.85615	*PsmiR* *390b* *-5p*	2.064972	1.580483	*PsmiR* *168a* *-3p*	−1.07857	2.107143
*PsmiR* *166h* *-3p*	4.76017	−1.62356	*PsmiR* *6108c*	2.019608	−2.28889	*PsmiR* *6441*	−1.11912	2.524147
*PsmiR* *165a*	4.607143	−2.93182	*PsmiR* *858b*	1.95122	−2.66667	*PsmiR* *5266*	−1.17936	1.447362
*PsmiR* *5054*	4.5	1.636364	*PsmiR* *162a*	1.95092	−1.58472	*PsmiR* *171a*	−1.18376	1.478632
*PsmiR* *5371* *-5p*	4.270833	−7.59259	*PsmiR* *6113*	1.923109	−1.81789	*PsmiR* *160a* *-3p*	−1.33333	1.088889
*PsmiR* *6478*	4.21875	−1.44385	*PsmiR* *172a*	1.882353	−1.14286	*PsmiR* *5658*	−1.69531	1.129688
*PsmiR* *5213* *-5p*	4.191126	−2.0264	*PsmiR* *167a*	1.87535	−1.51471	*PsmiR* *1881*	−1.95918	2
*PsmiR* *394a*	4.123779	−3.28831	*PsmiR* *2105*	1.797872	−1.67327	*PsmiR* *2916*	−2.56452	3.516129
*PsmiR* *169r* *-3p*	3.83945	−1.36098	*PsmiR* *398b* *-3p*	1.615385	1.857143	*PsmiR* *157a*	−3.38182	1.963636
*PsmiR* *5139*	3.761062	−3.57143	*PsmiR* *3630* *-3p*	1.585366	1.307692	*PsmiR* *156k*	−5.15686	3.156863
*PsmiR* *169a*	3.4	−2.20779	*PsmiR* *7984a*	1.581301	1.224936	*PsmiR* *5519*	2.25	0
*PsmiR* *168a*	3.218157	1.351579	*PsmiR* *6279*	1.571429	−0.29412			

Note: + and − indicate the induction and repression of miRNA, respectively.

The sequencing results showed that the abundance of novel miRNAs was relatively less than that of conserved miRNAs (Table 5). Among these 17 predicted novel miRNAs, *PsmiR1* was dramatically up-regulated during dormancy release. *PsmiR3* and *PsmiR4* were sharply reduced from 6 d to 18 d chilling treatments, *PsmiR9*, *PsmiR10* and *PsmiR13* showed opposite pattern with that of *PsmiR3* and *PsmiR4*. *PsmiR7*, *PsmiR*11, *PsmiR*12, *PsmiR15* and *PsmiR17* were detected only in flower buds after 18 d chilling treatments.

### Expression Validation of tree peony miRNAs

**Figure 7:**
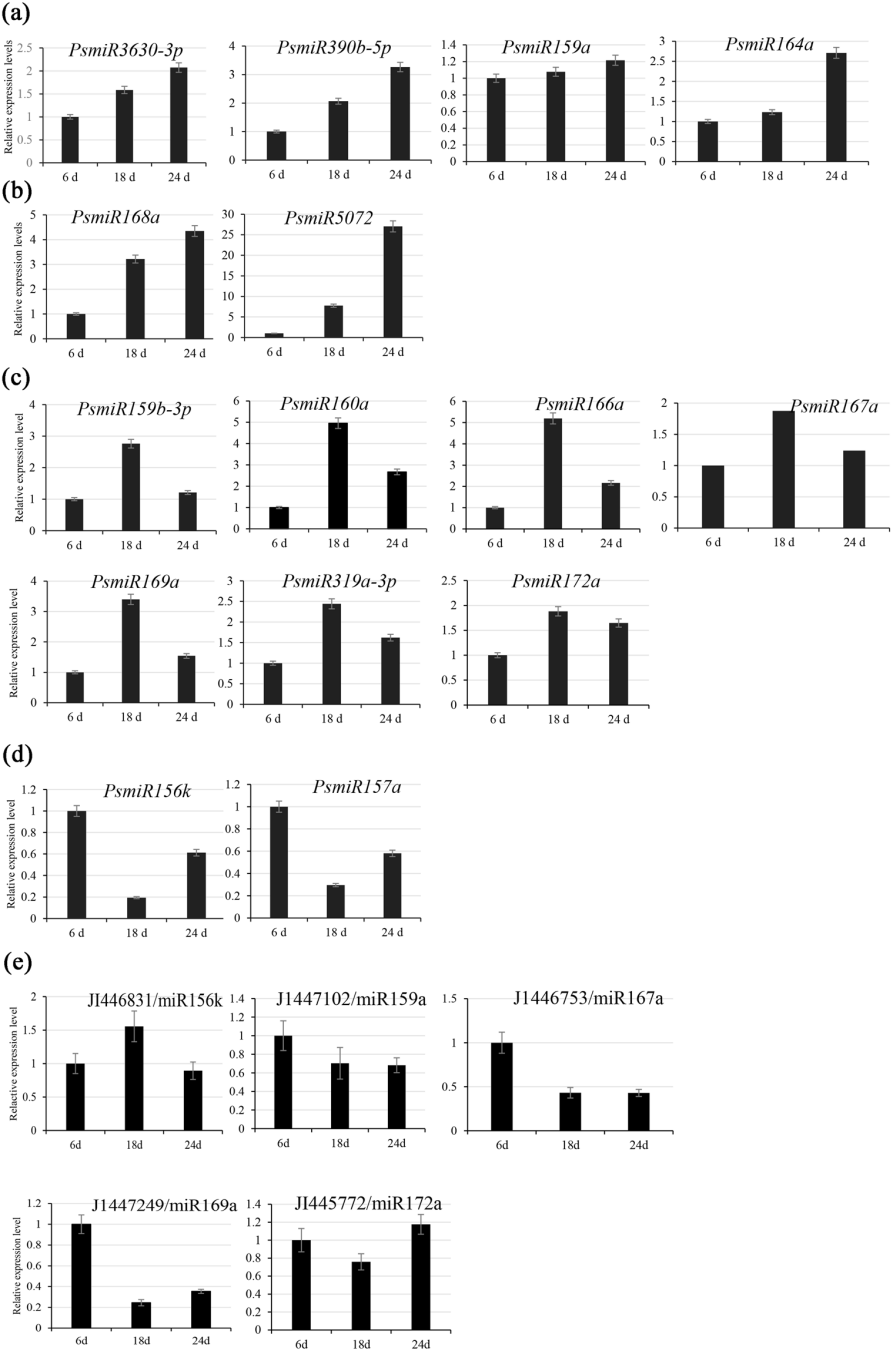
Validation of miRNAs expression patterns by Reverse transcriptase quantitative PCR (RT-qPCR) and expression patterns of partially corresponding target genes in our microarray results at three physiological stages. Type (**a**) slowly increased; type (**b**) suddenly increased; type (**c**) first increased and then decreased; type (**d**) first decreased and then increased. (**e**) Expression patterns of partially corresponding target genes in our microarray results.

To confirm the expression patterns of miRNAs, as well as detect their responses to chilling treatments at three physiological stages, the expression of 15 conserved miRNAs, whose sequencing counts altered significantly after treatment, were analyzed by RT-qPCR. It is showed that the expression levels of miRNAs were a constant change process with the time of treatment. We classified them into four types (Fig. 7). Type a: slowly increased (*PsmiR3630*, *PsmiR390b-5p*, *PsmiR159a* and *PsmiR164a*); type b: suddenly increased (*PsmiR168a* and *PsmiR5072*); type c: first increased and then decreased (*PsmiR159b-3p*, *PsmiR160a*, *PsmiR166a*, *PsmiR167a*, *PsmiR169a*, *PsmiR319-3p* and *PsmiR172a*); type d: first decreased and then increased (*PsmiR156k* and *PsmiR157a*). These results suggest that miRNAs belonging to type c were early stage response miRNAs, those belonging to type a and type b might accelerate endo-dormancy release. Notably, the expression trends of two *PsmiR159* family members, *PsmiR159a* and *PsmiR159b-3p* were different, indicating that the expression of miRNAs is a multiform process with the altered time of chilling treatments. Combining with our microarray data under same treatments^11^, five target genes of five miRNAs (miR156k, miR159a, miR167a, miR169a and miR172a) showed inverse expression patterns (Fig. 7e).

## Discussion

Tree peony is an important horticultural crop worldwide of great ornamental, medical and economic value. Native to China, tree peony is regarded as “King of flower” and have deep botanical history in Chinese culture. It is crucial to understand the molecular mechanism of dormancy, which is a main obstacle for tree peony forcing culture. Based on morphological changes of *Paoenia ostii* ‘Feng Dan’ and global mRNA expression profiling, the physiological status of flower buds receiving less than 18 d chilling treatment are regarded as endo-dormancy, and that receiving more than 18 d are defined as eco-dormancy^11^. miRNAs are paid more and more attention as key regulators of gene activity in animals and plants^26,38,39^. In this study, we adapt high-throughput sequencing technology to identify sRNAs from *Paeonia ostii* and analyze their response to dormancy release. This work will provide useful information to deepen our understanding of the miRNA regulatory mechanisms during dormancy release.

There are 243, 511 and 207 miRNAs annotated in Arabidopsis, rice and soybean according to the miRBase database, respectively^40–42^. In this study, we first completed construction of sRNA libraries (6 d, 18 d and 24 d chilling treatments) in tree peony and obtained over 19 million 16–30 nt reads. The size distribution of sRNAs revealed that 21, 22, 23 and 24 nt sRNAs were relatively abundant, of which 24 nt sRNAs were significantly higher than others. Similar results were observed from most plants, such as Arabidopsis, rice, tomato^43^, cucumber^44^, apple^45^, peach^46^ and rose^33^. However, 21 nt-long sRNAs were the second enriched in this study, which was different with previous reports in Arabidopsis (*Arabidopsis thaliana*) and rice (*Oryza sativa*)^30,47,48^. But in poplar, the 21 nt sRNAs are the most abundant^49^. Most of 21 nt sRNAs in our data started with uridine, which was consistent with the observation that *ARGONAUTE1* (*AGO1*) usually harbors miRNAs with a 5′ terminal uridine. 24 nt sRNAs had start-nucleotide preference of adenosine, which was also reported in previous work^26,27,29^.

Tree peony endo-dormancy transcriptome database was employed to predict putative targets of tree peony miRNAs. The well-known conserved miRNAs including *miR156*, *miR159*, and *miR164* have been identified. However, nearly half of known miRNAs and three novel miRNAs were not predicted homologous to any proteins in the Genbank nr database, which might because of the incomplete tree peony genome and limited number of transcript data in public database. Hundreds of miRNAs have been surveyed since high-throughput sequencing technology is widely used, but little has been done on identifying and analyzing miRNAs in tree peony and their response during dormancy release. Total 112 known miRNAs belonging to 99 families were identified in the three libraries. Two miRNAs families, *PsmiR159* and *PsmiR166*, were relatively abundant. *PsmiR166a* increased continuously until dormancy release (6 d –18 d) and had a very low expression level at eco-dormant stage (24 d), which was consistent with recent studies in poplar^34,50^. For the expression level of 17 novel miRNAs, *PsmiR1* was continuously up-regulated from dormancy to eco-dormancy stage, *PsmiR9, PsmiR10* and *PsmiR*13 (up-regulation) and *PsmiR3* and *PsmiR4* (down-regulation) had converse expression pattern at the early stage of dormancy release.

### Cold-responsive and auxin-related miRNAs

A continuous chilling accumulation is an effective natural or artificial way to release dormancy in tree peony. The endo-dormant bud can respond to chilling treatment, which stimulates growth-promoting respond signals including auxin or appropriate outside conditions (such as exogenous GA). In our study, there were 112 conserved miRNAs differentially expressed between 6 d and 18 d chilling library and between 18 d and 24 d chilling library. Among them, *PsmiR160a* was highly expressed in endo-dormancy release (18 d chilling) and quickly decreased in eco-dormancy (24 d chilling). *MiR160* targeted auxin response factor 10/16/17 (*ARF10*/*ARF16*/*ARF17*) and negatively regulated auxin signaling^51,52^. Ding *et al*. found that *miR160* was highly expressed in eco-dormancy (five weeks cold treatment)^34^. In our case, the high expression of *PsmiR160* in endo-dormancy release might because of the difference of dormancy mechanism exist in tree peony and poplar. On the other hand, the expression of *miR160* in endo-dormancy release may strengthen the auxin signal by inhibition of its target genes, and this hypothesis is also consistent with earlier report that exogenous GA could effectively promote endo-dormancy release^10^. Ding *et al*. reported other two auxin-related miRNAs, *miR390* and *miR167*, increased during active growth^34^. In our study, the expression of *PsmiR390b* was steadily increased during the transition from endo-dormancy to eco-dormancy, and *PsmiR167a* was significantly induced during dormancy release, which suggest that auxin signal pathway participated in the process of dormancy release. We also found that *PsmiR168a* was continuously up-regulated from endo-dormancy stage to eco-dormancy stage, the same trend was found in poplar^34^. Similarly, *miR168*, member of the *Csn-miR168* family, was found to be a cold-responsive miRNA, which was induced in two tea cultivars after 12 h of cold treatment^22^. *MiR168* regulates its target *ARGONAUTE1* (*AGO1*) to participate in miRNA biogenesis^37^. The high expression level of *PsmiR168a* would lead to the repression of *AGO1*, which would cause a reduction in the miRNA expression levels. The up-regulation of *PsmiR168a* suggested that cold-responsive miRNA participated in the release of endo-dormancy, their inductions were also consistent with its function of miRNA biogenesis^37^.

### MiRNA targets

Since the genome of *Paoenia ostii* is not publicly available, the mRNA transcriptome of *Paoenia ostii* flower buds^11^ were employed as a reference to predict the putative miRNA targets. Based on GO annotation, more than half of the predicted targets in tree peony were involved in binding, catalytic activity, metabolic process and cellular process. For example, *miR5141* targets gene encoding ATP synthase, which have been reported to be involved in ATP synthesis and ATP utilization during seed dormancy breaking^53^. In pear, specific enrichment of unigenes was observed for 15 pathways involved in metabolic processes including oxidative phosphorylation^4^. Several other target transcripts, which encode alpha N-terminal protein methyltransferase, Endoglucanase, GTPase-activating protein and F-box domain associated with various biological processes and cellular activities were also detected. For instance, *PsmiR395* targets genes encoding enzyme beta galactosidase, which recently have been reported to be involved in cell wall modification during the transition from dormancy to eco-dormancy in onion bulbs^54^. *PsmiR171* targets genes coding endoglucanase, which had been shown to be antifreeze proteins during seed germination in sunflower^55^. F-box proteins, the target of *PsmiR169*, have been identified previously as a key regulator of karrikin signaling and seed dormancy in Arabidopsis^56^. Novel miRNAs target genes were also predicted, but only two of them have not been found target relationship.

*MiR169* and *miR166* regulated cellular process and biological process by acting on their target genes. *MiR166* function mainly in vascular development^36^, and the down-regulation of *PsmiR166a* at eco-dormant stage might help to increase the expression level of its target gene. In addition, Potkar *et al*. found that *ptrmiR169a* and its target gene *PtrHAP2-5* showed inverse expression patterns during the dormancy period, which suggests that *miR169* mediate attenuation of the target *HAP2-5* transcript at this process^24^. Jeyaraj *et al*. found that *CsmiR169* targeted *COBRA*-like protein encoding gene and regulated cellulose synthase, which suggests that *miR169* have possible role in cell cycle and other biological function during the bud development^23^. In our study, *PsmiR169a* was highly expressed at the early stage of dormancy release and steadily down regulated at eco-dormant stage, and similar results were obtained during vegetative bud dormancy period of aspen^24^.

*MiR*15*6* and *miR172* regulate and control the juvenile-to adult vegetative transition by targeting transcription factors SQUAMOSA promoter-binding protein-like (*SPL*) and *APETALA2* (*AP2*) genes in both annual herbs^17,57^ and woody perennial plants^58^, showing converse expression patterns and regulatory relationships^57^. It is noteworthy that in our study we found the expression levels of *PsmiR156k* and *PsmiR172a* during the transition from endo-dormancy to eco-dormancy also had the converse expression patterns. Similarly, Ding *et al*. also found *miR156* and *miR172* showed completely converse expression patterns during the dormancy-active growth transition^34^. Our results showed that target genes (JI446524 and JI446831, putative *AP2* and *SPL* genes) had cleavage sits of *PsmiR172a* and *PsmiR156a*, respectively, which suggested that m*iR156* and *miR172* might play an important role during dormancy transition, which need to be further confirmed by experiments.

## Materials and Methods

### Plant materials

Four-year-old tree peony plants (*Paoenia ostii* ‘Feng Dan’) were potted and moved to refrigeration house with temperature 0–4 °C from 5 November to 30 December, 2014 in Qingdao, Shandong, China. The morphologic observation indicated flower buds receiving less than 18 d chilling treatment are in physiological status of endo-dormancy, while those receiving more than 18 d chilling treatment were in eco-dormancy physiological status^11^. Therefore, in order to decrease individuality, more than 5 plants were mixed buds-three buds for each individual were collected after 6 d, 18 d and 24 d chilling requirement fulfilling. Three replicates samples were harvested and immediately frozen in liquid nitrogen and stored at −80 °C until further use.

### Small RNA library construction and sequencing

Total RNA from tree peony flower buds after chilling treatments (6 d, 18 d and 24 d) was extracted using TRIzol reagent (Invitrogen, Carlsbad, CA, USA) following the manufacturer’s instruction and separated on 15% denaturing polyacrylamide gels. The 16–30 nt sRNAs were excised and recovered. The adapters (5′ and 3′) were ligated to the isolated sRNAs, which were sequentially reverse transcribed and amplified by PCR. The purified PCR products were sequenced using an Illumina Genome Analyzer (Illumina, USA) at Beijing Biomarker Technologies, Beijing, China.

### Analysis of sequencing data

Raw sequence reads were produced by Illumina Genome Analyzer at Biomarker-Beijing, China and processed into clean full length reads by the Biomarker small RNA pipeline. During this procedure, all low-quality reads including 3′ adapter reads and 5′ adapter contaminants were removed. The remaining high-quality sequences were trimmed of their adapter sequences. Sequences larger than 30 nt and smaller than 16 nt were discarded. All high-quality sequences were considered as significant and further analyzed.

All matched sRNA sequences were categorized into classes including miRNAs, siRNAs, ribosomal RNAs (rRNAs), tRNAs, snRNAs, snoRNAs, repeat-associated sRNAs and degrade tags of extrons of introns, etc. Then, the clean sequences were annotated by performing BLASTn searches against the Rfam (http://www.Sanger.ac.uk/Software/Rfam) and NCBI (http://blast.ncbi.nlm.nih.gov/Blast.cgi) databases, the detailed processes were following: the clean data were aligned with known miRNAs (miRNA precursors and mature miRNAs) registered in miRBase 21.0 (http://microran.sanger.ac.uk/sequence/index.html/) because of the difference among species, this process allowed two mismatches and free gaps to get temporary miRNA sequences and count of miRNA families; The highest-expression miRNA for each temporary mature miRNA family were selected to form a temporary miRNA database of *Paoenia ostii*. Finally, alignment of clean data to temporary miRNA database to identify conserved miRNAs in *Paoenia ostii*, only those perfect matching (≤two mismatches) were considered as conserved miRNAs. Potential novel miRNAs were identified using criteria that were previously developed for plant miRNA prediction^59^. The unique fold back structures of miRNA precursors can be utilized to predict novel miRNAs using MIREAP program (http://sourceforge.net/projects/mireap/). Potential targets for both known and novel miRNAs were identified on TAPIR and Target Finder based on *Paeonia ostii* transcriptome sequencing data^10^ according to the search algorithm that only three or fewer mismatches and no gap are allowed to be present in the complementarily between miRNAs and their corresponding targets^32^. The biological function category of the predicted targets was annotated against the Universal Protein Resource (http://www.uniprot.org).

### Differential expression analysis of miRNA and Reverse Transcriptase quantitative PCR (RT-qPCR) and 5′ RLM-RACE

Differential expression analysis of miRNAs was performed based on sequence reads generated from three libraries after different chilling treatments according to the method described by Ren^49^. In detail, the expression of miRNAs was normalized to obtain the number of miRNAs per million reads [normalized expression = (number of miRNA reads/total number of clean reads) × 1,000,000]. Normalized miRNA reads with values less than one in three libraries were excluded. The remaining miRNAs were used to calculate differences in expression by fold change (normalized miRNA reads in 18 d or 24 d chilling treatment/normalized miRNA reads in 6 d chilling treatment) and significant *P*-values^60,61^.

To validate miRNA expression, sRNAs were isolated from flower buds after different chilling treatments using an RNAiso for small RNA (TaKaRa, Dalian, China) following the manufacturer’s instructions. Then, the sRNA was polyadenylated by poly (A) polymerase, and first-strand cDNA was obtained using SYBR^®^ Primescript miRNA RT-PCR Kit (TaKaRa, Dalian, China). Briefly, the polyA was added to the 3′ of total RNA, then the RNA was reverse-transcribed with an oligo-dT adaptor. Quantitative PCR was performed in a total volume of 25 μL, containing 2 μL cDNA, 0.4 μM PCR forward primer (1 μL), 0.4 μM Uni-miR RT-qPCR primer (1 μL), 12.5 μL of 2 × SYBR premix Ex Taq ΙI, and 8.5 μL dd H_2_O. The reactions were completed using Roche Light Cycler 480 (Roche, Mannheim, Germany) with the following program: 95 °C for 10s and 40 cycles of 95 °C for 5s, 55 °C for 30s and 72 °C for 15s. The reactions were run in triplicate and the 2^−ΔΔCt^ relative quantification method was used to calculate the relative changes in gene expression^62^. Small nuclear RNA U6 was used as endogenous reference, primers used in this study were listed in Supplementary Table S1.

To conform whether the predicted targets were cut by miRNAs and cleavage sites, the 5′ RLM-RACE were carried out using the FirstChoice RLM-RACE Kit (Ambion). Specifically, one microgram total RNA was firstly ligated to 5′ RACE oligo adaptor without calf intestine alkaline phosphatase and tobacco acid pyrophosphatase treatments. Then, the ligated RNA was used to synthesize the cDNA. The primers of *miR172a* target gene (JI446524) (5′-TCGGAGAAATGCTTTGTCCATGGCCAT-3′) and* miR156a* target gene (JI446831) (5′-TTGCGAGGTTCTGGGTTTGGAG-3′) for 5′ RLM-RACE were designed by Primer premier 5.0 software (Supplementary Table S1). PCR was carried out according to the manufacturer instructions, and the PCR products were purified by 1.0% agarose gel electrophoresis and cloned into the pMD18-T vector (Takara, Dalian, China) for sequencing.

### Availability of Data and Materials

Our data have been presented in the main paper or additional supporting files.

## Electronic supplementary material

Supplementary information Figure S1

Table S1

Additional file 1

Additional file 2

Additional file 3

Additional file 4
