# Supplementary material for: Identification and characterization of microRNAs in tree peony during chilling induced dormancy release by high-throughput sequencing
Source: Sci Rep. 2018 Mar 14;8:4537. doi: 10.1038/s41598-018-22415-5 (PMC5852092; doi:10.1038/s41598-018-22415-5)
Supplement: Supplementary file 1 — Supplementary information Figure S1 [file 41598_2018_22415_MOESM1_ESM.docx]

**Supplementary information**

Identification and characterization of microRNAs in *Paoenia suffruticosa* during chilling induced dormancy release by high-throughput sequencing

Yuxi Zhang^¶^, Yanyan Wang^¶^, Xuekai Gao, Chunying Liu, Shupeng Gai^*^

College of Life Sciences, Qingdao Agricultural University, Key Lab of Plant Biotechnology in Universities of Shandong Province, Changcheng Road 700, Qingdao, China

^¶^These authors contributed equally to this work

^*^ Corresponding author: Shupeng Gai: [spgai@qau.edu.cn](mailto:spgai@qau.edu.cn)


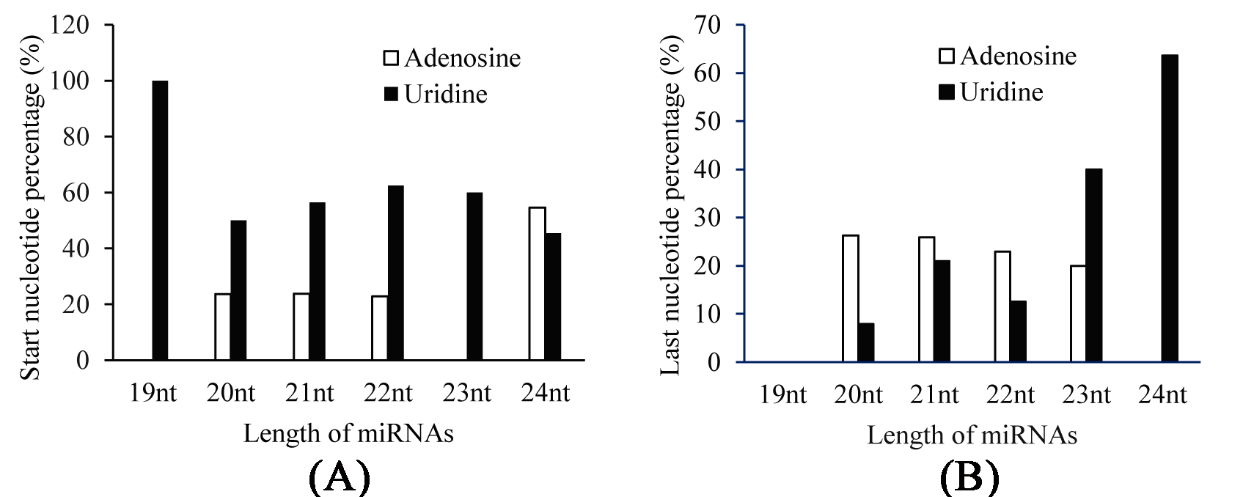


1. (b)

S1 Fig Nucleotide preference of 427 Arabidopsis small RNAs deep sequencing datasets from miRBase database. (a) Percentage of adenosine or uridine at the start position of 19 to 24-nucleotide (nt) small RNAs. (b) Percentage of adenosine or uridine at the last position of 19 to 24-nucleotide (nt) small RNAs.
