# Supplementary material for: Identification and characterization of microRNAs in tree peony during chilling induced dormancy release by high-throughput sequencing
Source: Sci Rep. 2018 Mar 14;8:4537. doi: 10.1038/s41598-018-22415-5 (PMC5852092; doi:10.1038/s41598-018-22415-5)
Supplement: Supplementary file 2 — Table S1 [file 41598_2018_22415_MOESM2_ESM.docx]

**Supplementary information**

Identification and characterization of microRNAs in *Paoenia suffruticosa* during chilling induced dormancy release by high-throughput sequencing

Yuxi Zhang^¶^, Yanyan Wang^¶^, Xuekai Gao, Chunying Liu, Shupeng Gai^*^

College of Life Sciences, Qingdao Agricultural University, Key Lab of Plant Biotechnology in Universities of Shandong Province, Changcheng Road 700, Qingdao, China

^¶^These authors contributed equally to this work

^*^ Corresponding author: Shupeng Gai: [spgai@qau.edu.cn](mailto:spgai@qau.edu.cn)

| Table S1. Reverse transcript PCR (RT-PCR), reverse transcriptase quantitative PCR (RT-qPCR) and 5’RLM-RACE primers used in this study | | |
| --- | --- | --- |
| Primer name | Primer sequences (5’-3’) | Tm (°C) |
| *PsmiR*3630-3p | TGGGAATCTCTCTGATGCAC | 55 |
| *PsmiR*390b-5p | AAGCTCAGGAGGGATAGCACC | 58 |
| *PsmiR*159a | TTTGGATTGAAGGGAGCTCTA | 55 |
| *PsmiR*164a | TGGAGAAGCAGGGCACGTGCA | 60 |
| *PsmiR*168a | TCGCTTGGTGCAGGTCGGGAA | 60 |
| *PsmiR*5072 | AACGACTCCCCAGCAGAGTCGCCA | 60 |
| *PsmiR*159b-3p | TATTGGAGTGAAGGGAGCTCC | 55 |
| *PsmiR*160a | TGCCTGGCTCCCTGTATGCCA | 60 |
| *PsmiR*166a | TCGGACCAGGCTTCATTCCCC | 60 |
| *PsmiR*167a | TGAAGCTGCCAGCATGATCTGA | 60 |
| *PsmiR*169a | CAGCCAAGGATGACTTGCCGA | 60 |
| *PsmiR*319a-3p | TTGGACTGAAGGGAGCTCCC | 55 |
| *PsmiR*172a | AGAATCTTGATGATGCTGCAT | 55 |
| *PsmiR*156k | TGACGGAGAGAGAGAGCACAC | 55 |
| *PsmiR*157a | TTGACAGAAGATAGAGAGCAC | 55 |
| *PsmiR1* | AGGGACTCCTTTCACTCCACT | 60 |
| *PsmiR3* | GGTGGACTGCTCGAGCC | 60 |
| *PsmiR4* | TATGAGACTTGGACGAGGCAC | 60 |
| *PsmiR9* | CGGTGGACTGCTCGAGCCG | 60 |
| *PsmiR13* | TTGTTTGAATTCTTGCAACAGA | 55 |
| *PsU6* | GCACAAATCGAGAAATGGTCCAAATCC | 55-60 |
| JI446524-RLM | TCGGAGAAATGCTTTGTCCATGGC | 65 |
| JI446831-RLM | TTGCGAGGTTCTGGGTTTGGAG | 65 |
